# Cigarette Smoke Impairs Airway Epithelial Wound Repair: Role of Modulation of Epithelial-Mesenchymal Transition Processes and Notch-1 Signaling

**DOI:** 10.3390/antiox11102018

**Published:** 2022-10-12

**Authors:** Serena Di Vincenzo, Dennis K. Ninaber, Chiara Cipollina, Maria Ferraro, Pieter S. Hiemstra, Elisabetta Pace

**Affiliations:** 1 Institute of Translational Pharmacology (IFT), National Research Council of Italy (CNR), Via Ugo La Malfa, 153, 90146 Palermo, Italy; 2 Department of Pulmonology, Leiden University Medical Center, 2333 ZA Leiden, The Netherlands; 3 Ri.Med Foundation, Via Bandiera, 11, 90133 Palermo, Italy

**Keywords:** cigarette smoke, proximal airways, repair processes, epithelial-mesenchymal transition, TGF-beta1, Notch-1 pathway

## Abstract

Cigarette smoke (CS) induces oxidative stress and chronic inflammation in airway epithelium. It is a major risk factor for respiratory diseases, characterized by epithelial injury. The impact of CS on airway epithelial repair, which involves epithelial-mesenchymal transition (EMT) and the Notch-1 pathway, is incompletely understood. In this study, we used primary bronchial epithelial cells (PBECs) to evaluate the effect of CS on epithelial repair and these mechanisms. The effect of CS and/or TGF-beta1 on wound repair, various EMT and Notch-1 pathway markers and epithelial cell markers (TP63, SCGB1A) was assessed in PBECs cultured submerged, at the air–liquid interface (ALI) alone and in co-culture with fibroblasts. TGF-beta1 increased epithelial wound repair, activated EMT (shown by decrease in E-cadherin, and increases in vimentin, SNAIL1/SNAIL2/ZEB1), and increased Notch-1 pathway markers (NOTCH1/JAGGED1/HES1), MMP9, TP63, SCGB1A1. In contrast, CS decreased wound repair and vimentin, NOTCH1/JAGGED1/HES1, MMP9, TP63, SCGB1A1, whereas it activated the initial steps of the EMT (decrease in E-cadherin and increases in SNAIL1/SNAIL2/ZEB1). Using combined exposures, we observed that CS counteracted the effects of TGF-beta1. Furthermore, Notch signaling inhibition decreased wound repair. These data suggest that CS inhibits the physiological epithelial wound repair by interfering with the normal EMT process and the Notch-1 pathway.

## 1. Introduction

Cigarette smoke is the major risk factor for respiratory diseases which are commonly characterized by damage to the respiratory epithelium [1,2]. This damage can be directly caused by exposure to toxic substances present in cigarette smoke, but also indirectly by reactive oxygen species (ROS) produced by lung cells resulting in oxidative stress, and by the chronic inflammation triggered by cigarette smoke [3,4,5]. The airway epithelium plays a major role in lung health by providing a barrier to the environment, maintaining the normal airway structure and function, and contributing to host defenses [6]. Different mechanisms that initiate epithelial repair are activated when the epithelial barrier of the airways is damaged. Activation of these mechanisms leads to migration, proliferation and subsequent differentiation of the basal cells, ultimately resulting in restoration of the pseudostratified structure characteristic of the airway epithelium [7]. 

A biological process involved in both physiological and pathological repair and fibrosis is the epithelial-mesenchymal transition (EMT) [8]. During EMT, epithelial cells undergo complex reprogramming through which they lose epithelial characteristics and acquire a mesenchymal phenotype [9,10]. EMT occurs in three distinct biological settings and the mechanisms of induction and progression of EMT vary markedly from one setting to another [9,11,12]. EMT, that is associated with implantation, embryogenesis and organ development, is classified as type 1 EMT, whereas type 2 EMT occurs with wound healing, tissue regeneration, and organ fibrosis. In this case, the EMT program begins as part of an event associated with repair following damage and inflammation, but continuous triggering of EMT due to, e.g., persistent inflammation may lead to tissue fibrosis. Finally, type 3 EMT is caused by genomic alterations acquired by tumor cells and provides tumor cells with invasive properties.

Type 2 EMT is an essential mechanism for physiological tissue repair. Wound healing consists of several stages, starting with an inflammatory response induced by external insults that is associated with cell proliferation, migration and remodeling of the extracellular matrix [8,11,12]. Oxidative stress plays a role in the regulation of normal wound repair. However, excessive and chronic oxidative stress dysregulates inflammation, resulting in a delayed repair of the damage, that has been shown to be reversed by antioxidant molecules [13]. During wound repair, a complex signaling network is activated involving numerous growth factors, which are also known to be involved in the initiation and regulation of EMT. The finding that fibroblast growth factor (FGF), epidermal growth factor (EGF), hepatocyte growth factor (HGF) and transforming growth factor-beta (TGF-beta) are growth factors that are activators of both processes [8,11,12] is in line with the role of EMT in restoring barrier integrity following injury. Furthermore, myofibroblasts, the major players in the remodeling and maturation phase of wound healing, may also arise from resident epithelial cells that have transformed via EMT [8,14]. However, during physiologic wound healing, myofibroblasts undergo apoptosis once damage repair is complete, continued activation and decreased death of myofibroblasts results in the formation of fibrotic tissue. Such an aberrant EMT and wound repair has been linked to the dysregulation of EMT triggered by persistent insults [8,15]. Importantly, TGF-beta1, one of the main regulators of both EMT and physiologic wound healing, is also involved in fibrosis processes. 

Another pathway playing a role in the wound healing mechanism is Notch signaling [16]. The Notch pathway is involved in a variety of differentiation/development and tissue repair/homeostasis events [16,17] and in EMT both in normal tissue, during the processes of embryogenesis and cell differentiation, and in different types of carcinomas such as breast, lung, pancreas and squamous cell carcinomas [11,18,19,20,21]. Notch activates EMT by regulating the expression of transcription factors implicated in EMT, such as SNAIL1 and SNAIL2, which repress the transcription of the E-cadherin gene and induce the expression of mesenchymal markers, such as fibronectin and vimentin [11,22,23,24]. 

Notch proteins are members of a family of type 1 transmembrane receptors. The binding of the receptor to the ligand induces Notch cleavage and release of the intracellular portion (NICD). NICD moves into the nucleus and induces the transcription of specific target genes including HES1, 2, and 5, and HEY1, 2, and L [16,25]. It has been shown that the expression of components of the Notch pathway is downregulated in the airway epithelium of smokers with chronic obstructive pulmonary disease (COPD) [26]. In this regard, using a human bronchial epithelial cell line, it has been demonstrated that cigarette smoke extract reduces Notch-1 protein nuclear expression [27].

Growing evidence has shown that chronic exposure to cigarette smoke induces an uncontrolled oxidative stress and a chronic inflammatory status affecting the repair process [5,13,28,29], a decrease of Notch target genes and morphological changes in the epithelium of the small airways such as basal cell hyperplasia, destruction of the cilia, and secretory cell hyperplasia with increased goblet cell hyperplasia [30,31,32]. The mechanisms involved in these processes are still not completely understood. 

The aim of the present study was to assess whether cigarette smoke interfered with repair processes and whether this is linked to dysregulation of EMT and Notch signaling. We first used a simple in vitro model with undifferentiated primary human bronchial epithelial cells (PBECs) that were cultured submerged, to explore the effects of cigarette smoke on wound repair and EMT, and the involvement of Notch-1 pathway. Next, key findings were confirmed and extended using a more complex experimental model that reproduces a pseudostratified lung epithelium characterized by the presence of the different cell types present in the airway epithelium in situ. This was achieved by culturing PBECs at the air–liquid interface (ALI). Furthermore, we added human fibroblasts to ALI cultures, since crosstalk between fibroblasts and epithelial cells plays a role in the repair process. 

## 2. Materials and Methods

### 2.1. Human Primary Bronchial Epithelial Cells (PBECs) Culture

Human primary bronchial epithelial cells (PBECs) were isolated from macroscopically normal lung tissue obtained from patients undergoing resection surgery for lung cancer at the Leiden University Medical Center, the Netherlands. Patients from which this lung tissue was derived were enrolled in the biobank via a no-objection system for coded anonymous further use of such tissue (www.coreon.org, accessed on 15 August 2022). However, since 29 November 2020, patients are enrolled in the biobank using active informed consent in accordance with local regulations from the LUMC biobank with approval by the institutional medical ethical committee (B20.042/Ab/ab and B20.042/Kb/kb). Individual informed consent is not needed for the no-objection system. 

The PBECs used for the study derived from 11 non-current smokers and non-COPD donors [Gender (M/F): 5/6; Age: 63 ± 9; Never smokers/ex-smokers (non-smokers for more than 2 years): 3/8; no COPD: 11]. The isolation of PBECs from lung tissue was performed as follows: the bronchus ring was transferred to a solution with protease XIV and incubated for 2 h at 37 °C. Next, cells were carefully scraped from the inside of the ring, washed and seeded on 6-well plates which were pre-coated with a coating solution composed of human fibronectin (Promocell, Heidelberg, Germany), Bovine Albumin Fraction V (Thermo Fisher Scientific, Waltham, MA, USA) and PureCol (Advanced BioMatrix, Carlsbad, CA, USA). The isolated cells were cultured for at least one week in complete Keratinocyte Serum-Free Medium (KSFM) (Gibco, Thermo Fisher Scientific, MA, USA).

In the first set of experiments, PBECs were cultured submerged (sub-PBECs) to assess markers and processes in a less complex in vitro model. In brief, 30,000 cells at passage 2 were seeded on 6-well plates which were coated with the coating solution described. Cells were cultured in Bronchial Epithelial Cell Medium-basal (BEpiCM-b; ScienCell, Sanbio, Uden, The Netherlands) and Dulbecco’s modified Eagle’s medium (DMEM) (Stemcell Technologies, The Netherlands) (1:1 mixture), including 12.5 mM Hepes, 1 mM Glutamax (Gibco, Thermo fisher scientific, Waltham, MA, USA), bronchial epithelial cell growth supplement (BEpiCGS; Sciencell, Sanbio, The Netherlands), 100 U/mL penicillin and 100 μg/mL streptomycin (all from ScienCell, Sanbio, The Netherlands). When the cells reached confluence, the medium was changed to starvation medium (the same medium without the bronchial epithelial supplements) and after 24 h the cells were treated with 5 ng/mL of recombinant Human TGF-beta1 protein (R&D systems, Minneapolis, MN, USA) and/or with cigarette smoke extract and, in selected experiments with N-[N-(3,5-Difluorophenacetyl-L-alanyl)]-S-phenylglycine t-butyl ester (DAPT) (Santa Cruz Biotechnology, Dallas, TX, USA), a gamma-secretase inhibitor that blocks Notch signaling. The samples were harvested after 6 and 24 h or used for the wound healing assay.

For subsequent experiments, PBECs were cultured at the air–liquid interface (ALI-PBECs) for 14 days to achieve mucociliary differentiation as previously described [30,33]. In brief, 40,000 cells at passage 2 were seeded on 0.4 μm pore sized 12-well transwell membranes (Corning Costar, Glendale, AZ, USA) which had been coated with the coating solution, in the complete BEGM/DMEM medium as described elsewhere in this section. Cells were cultured submerged until confluence in complete medium with 1 nM of the retinoic receptor agonist EC23 (Tocris, Bristol, UK); next, the apical medium was removed and cells were differentiated by culture at ALI for 14 days in the same medium in which the concentration of EC23 was increased to 50 nM. Trans-epithelial electrical resistance [TEER > 500 Ω∙cm^2^], cilia beating and mucus secretion were assessed as markers of differentiation.

After 24 h, differentiated ALI-PBECs were stimulated with 5 ng/mL of TGF-beta1 added to the basal compartment and/or with whole cigarette smoke, and the samples were harvested after 6 and 24 h.

### 2.2. Fibroblast and PBEC Co-Culture 

MRC5 human lung fibroblasts (CCL-171; ATCC, Wesel, Germany DE) were cultured in Minimum Essential Medium (MEM) medium supplemented with 10% fetal bovine serum (FBS), 1 mM Glutamax (Gibco, Thermo Fisher Scientific, Waltham, MA, USA), 100 U/mL penicillin and 100 μg/mL streptomycin (ScienCell, Sanbio), and non-essential amino acids (NEAAS) (Invitrogen, Thermo Fisher Scientific) at 37 °C with 5% CO_2_. 25,000 cells were seeded in wells of a 12-well plate. Once confluence was reached, the cells were detached and seeded again on the bottom of a well of a transwell plate (Fb) or on lower half of the insert membrane (Fi) which on the upper half contained differentiated ALI-PBECs. The co-culture was maintained in the medium used for the ALI-PBECs. After two days of co-culture, the cells were stimulated as previously described.

### 2.3. Cigarette Smoke Extract (CSE) Stimulation

Cigarette smoke extract (CSE) was generated by using 3R4F research cigarettes (University of Kentucky, Lexington, KY, USA). Two cigarettes without filters were smoked by bubbling air in 2 mL of phosphate buffered saline (PBS) (Euroclone) using a linear pump. The smoke solution thus obtained was diluted 10 times and measured continuously with a spectrophotometer from a wavelength of 200 to 350 nm. The maximum optical density (OD) obtained was recorded and this was used to calculate the initial total arbitrary units (AU) of this solution using the following formula [34]:

ODmax × 2 × dilution factor = AU/mL.

Submerged PBECs were exposed to CSE 2 AU/mL diluted in medium for 30 min, followed by washing of the cells with PBS, and addition of fresh medium.

### 2.4. Whole Cigarette Smoke (WCS) Stimulation

ALI-PBECs alone or co-cultured with MRC5 fibroblasts were exposed to freshly generated whole cigarette smoke (WCS) using 3R4F cigarettes (University of Kentucky, Lexington, KY, USA), as previously described [30]. Briefly, cells were exposed in modified hypoxic chambers for 4–5 min to cigarette smoke derived from one cigarette, or to room air (Control); next, smoke was removed by ventilation with air over 10 min and cells were subsequently returned to the incubator. Cells were harvested for analysis after 6 and 24 h.

### 2.5. Wound Healing Assay

PBECs were seeded in a 6-well plate and were cultured submerged in medium to confluence. Three circular wounds were prepared in each well using a 200-µL pipette tip. After washing with PBS to eliminate debris resulting from creating the wound, cells were allowed to recover for one hour and then exposed to 5 ng/mL TGF-beta1, CSE 2 AU/mL and 10 μM DAPT. Digital images were acquired using a camera connected to an inverted phase-contrast light microscope at 0 h, 24 h and 48 h after wounding. The surface of the wound area was measured using the ImageJ software in order to assess remaining wound size and wound closure rates. For each wound, the wound area at 0 h was considered as 100%, and assessment of the area of the same wound after 24 h (T24 h) and 48 h (T48 h) of the same sample was used to calculate the residual wound. Results were expressed as percentage of area reduction at T24 h and T48 h compared to T0 h.

### 2.6. Immunofluorescence by Confocal Microscopy

Prior to fixing the cells, the transwell membranes with cells were rinsed with PBS. Next, 4% paraformaldehyde in PBS was added into the basal and apical compartments for 30 min at room temperature. Membranes were washed twice with PBS and ice-cold methanol was added for 10 min at 4 °C. Non-specific bindings sites were blocked by incubation in a solution of PBS with 1% (*w/v*) bovine serum albumin (BSA) and 0.3% (*v/v*) Triton-X-100 (PBT, Zwijndrecht, The Netherlands) for 30 min, then the insert was cut in four parts. One part was stained for E-cadherin (mouse IgG2a anti-E-cadherin; #610182; BD transduction, Franklin Lakes NJ, USA). After washing, the membrane was stained with anti-mouse secondary antibodies and 4′,6-diamidino-2-phenylindole (DAPI, 1:50, Sigma-Aldrich, Zwijndrecht, The Netherlands) in the dark for 30 min at room temperature. Next, membranes were put on a glass slide together with prolong gold anti fading reagent (Gibco, Thermo Fisher Scientific, Waltham, MA, USA) and covered with a cover glass. Slides were viewed by a Leica TCS SP8 confocal microscope (Leica Microsystems, Wetzlar, DE, Germany) at 630× original magnification.

Another part of the insert was stained for Vimentin (mouse IgG1 anti-vimentin (cloneV9); Dako, Denmark) and then with the specific secondary antibody and DAPI. The membrane was mounted on a slide as previously described. Images were acquired using the Operetta CLS system (PerkinElmer, Waltham, MA, USA).

Fluorescence intensity of the E-cadherin and Vimentin staining was measured using ImageJ software.

### 2.7. Gene Expression Evaluation

Gene expression of E-cadherin (CDH1), vimentin (VIMENTIN), SNAIL 1, SNAIL 2, Zinc finger E-box-binding homeobox 1 (ZEB1), Tumor protein 63 (TP63) and Secretoglobin family 1A member 1 (SCGB1A1) was evaluated using the following SYBR green protocol. Cells were harvested and total RNA was isolated using Maxwell^®^ 16 simply RNA tissue kit (Promega, Madison, WI, USA) following the manufacturer’s instruction. 1 μg mRNA was reverse-transcribed into cDNA using Oligo d(T). Relative gene expression compared to reference genes 60S ribosomal protein L13a (RPL13A) and mitochondrial ATP synthase subunit beta (ATP5B) were calculated according to the standard curve method. Reference genes were selected using the “Genorm” software (Genorm; Primer Design, Southampton, UK). Primer pairs are presented in Table 1. 

Gene expression of Neurogenic locus notch homolog protein 1 (NOTCH1), Jagged-1 (JAG1) and transcription factor HES-1 (HES1) were evaluated using the following protocol. Total RNA was extracted using TRIzol Reagent (Invitrogen, Thermo fisher scientific) following the manufacturer’s instruction. The equivalent volume to 1 μg RNA was reverse-transcribed to cDNA, using the iScript cDNA Synthesis kit (Bio-Rad, Hercules, CA, USA). Real-time quantitative PCR was carried out on Step One Plus Real-time PCR System (Applied Biosystems, Foster City, CA, USA) using specific FAM-labeled probes and primers (prevalidated TaqMan Gene expression assay for NOTCH1 Hs01062014-m1, JAG1 Hs01070032-m1, HES1 Hs00172878-m1, Applied Biosystems, Thermo fisher scientific). Gene expression was normalized to GAPDH (prevalidated TaqMan Gene expression assay for GAPDH, Hs03929097g1, Applied Biosystems), that was used as a reference gene. Relative quantification of mRNA was carried out with the comparative CT method (2^−ΔΔCT^) and was plotted as a relative fold-change compared to untreated cells that were chosen as the reference sample.

### 2.8. Statistics

Data were expressed as mean ± SD and analyzed by using Mann–Whitney and Wilcoxon test. At *p* value < 0.05, differences were considered to be statistically significant.

## 3. Results

### 3.1. Cigarette Smoke Reduces Epithelial Wound Repair: Impact of EMT Mechanism in Submerged-PBECs

**Figure 1:**
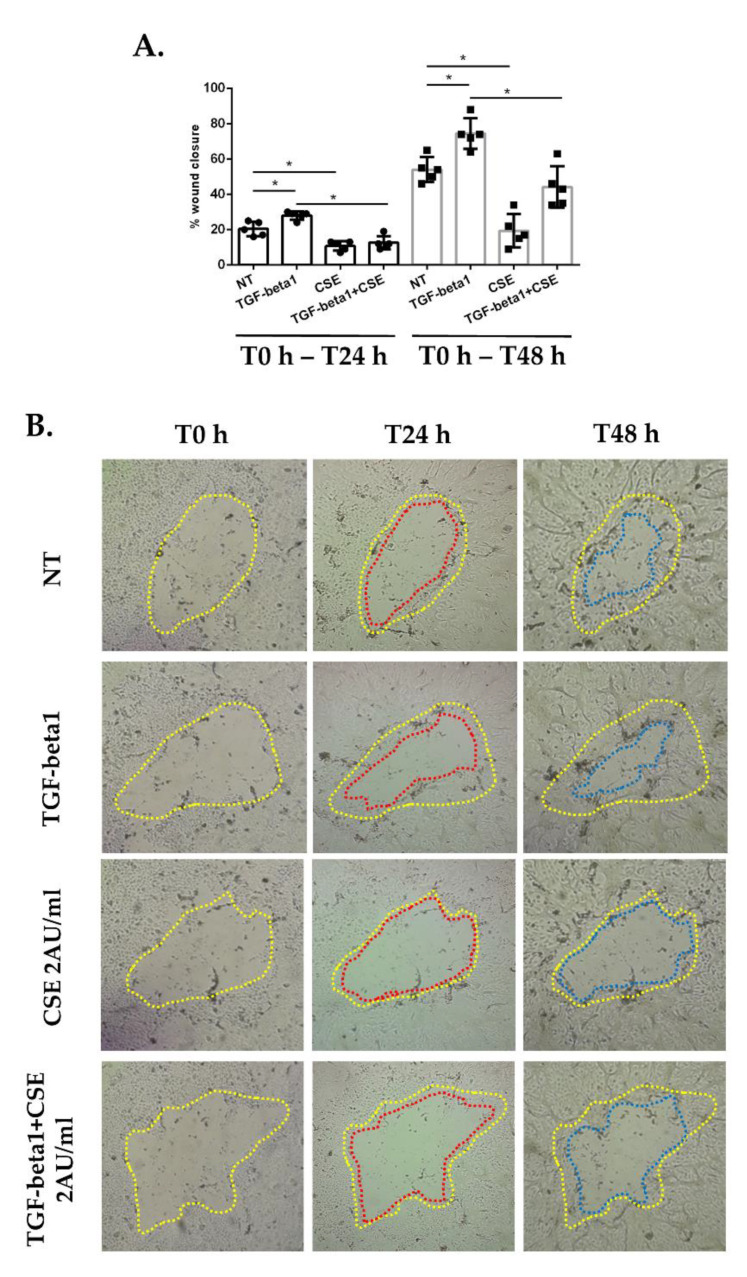
**Wound repair assay in submerged-PBECs.** (**A**,**B**) Sub-PBECs were seeded in a 6-well plate and were cultured to confluence. Three wounds were made in each well using a 200-µL pipette tip, and next sub-PBECs were stimulated with TGF-beta1 and CSE, and wound repair was monitored. (**A**) Results are reported as a percentage of area reduction at T24 h and 48 h compared to T0 h (*n* = 5). * *p* < 0.05 Mann–Whitney; and (**B**) Representative images from one donor. The line colors indicate: yellow, the area of the wound at T0 h; red, the area at T24 h; blue, the area at T48 h.

At the start of this study, we evaluated the effect of cigarette smoke in comparison to the effect of TGF-beta1, the main and most studied activator of EMT processes, on wound repair. We used a wound repair assay to measure the ability of the cells to repair a wound that was created in a monolayer of confluent cells. Submerged PBECs were exposed to TGF-beta1 or CSE 2 AU/mL, and wound repair was evaluated after 24 h and 48 h. The results showed that TGF-beta1 treatment increased wound repair, whereas exposure to CSE caused a significant decrease in wound closure (Figure 1A,B). In addition, we investigated whether cigarette smoke could interfere with the normal repair mechanism initiated by TGF-beta1, so we treated PBECs with the combination of CSE and TGF-beta1. The results showed that damage repair was slower when CSE was added in addition to TGF-beta1 compared to TGF-beta1 alone (Figure 1A,B).

**Figure 2:**
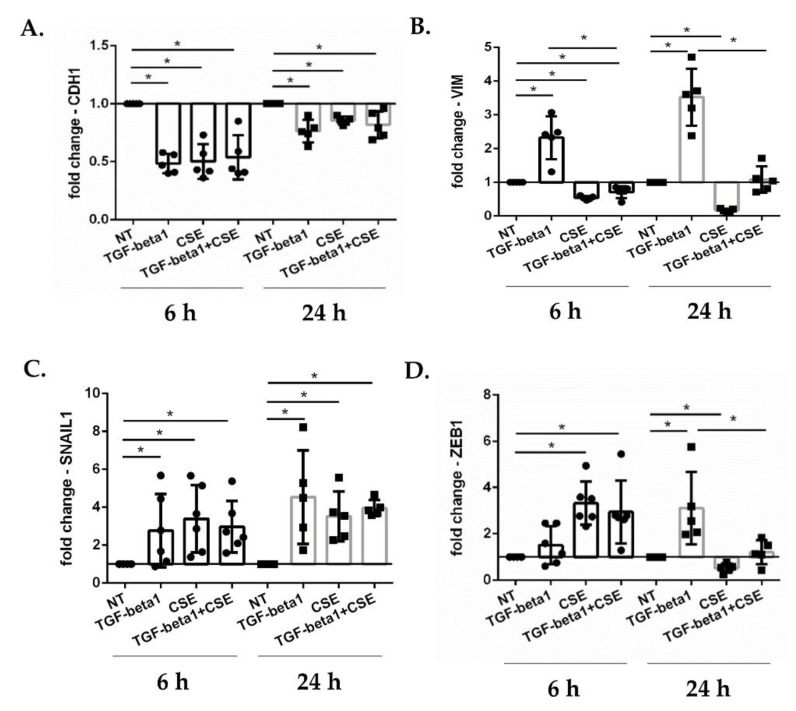
**CDH1, VIMENTIN, SNAIL1, ZEB1 gene expression in submerged-PBECs.** Sub-PBECs were stimulated with TGF-beta1 and/or CSE. After 6 h and 24 h, total RNA was extracted to evaluate CDH1 (**A**); VIMENTIN (VIM) (**B**) SNAIL1 (**C**); and ZEB1 (**D**) gene expression. Results from 5 independent donors are reported as mean fold change compared to not-treated (NT) ± SD (*n* = 5). * *p* < 0.05 Wilcoxon test.

To investigate why cigarette smoke slowed epithelial repair and study possible modulation of EMT, we evaluated how exposure to CSE changed the gene expression of major players in the EMT process: E-cadherin, vimentin, SNAIL1 and ZEB1. Submerged PBECs were exposed to TGF-beta1 and/or CSE 2 AU/mL, and gene expression of CDH1, VIMENTIN, SNAIL1 and ZEB1 after 6 and 24 h were evaluated by RT-PCR. The results showed that TGF-beta1 reduced CDH1 (Figure 2A) and increased VIMENTIN (Figure 2B) gene expression after 6 and 24 h of exposure. In contrast, whereas like TGF-beta1, CSE also reduced CDH1 (Figure 2A), in contrast to TGF-beta1 it reduced VIMENTIN (Figure 2B); in combination with TGF-beta1, the inhibitory effect of CSE on vimentin expression dominated (Figure 2B). Focusing on the EMT transcription factors, our results showed an increase of SNAIL1 (Figure 2C) and ZEB1 (Figure 2D) expression at 6 and 24 h caused by TGF-beta1 exposure, confirming the role of TGF-beta as activator of the EMT process. CSE also induced an increase of the expression of these two genes after 6 h of exposure, but after 24 h the increase in SNAIL1 expression persisted (Figure 2C), whereas ZEB1 was reduced (Figure 2D). Following exposure of the submerged PBECs to the combination of TGF-beta1 and CSE, CSE was found to reduce the stimulatory effect of TGF-beta1 on ZEB1 expression (Figure 2D), in line with the findings on vimentin.

### 3.2. Cigarette Smoke Reduces Epithelial Wound Repair: Impact of Inhibition of Notch Signaling in Submerged-PBECs

**Figure 3:**
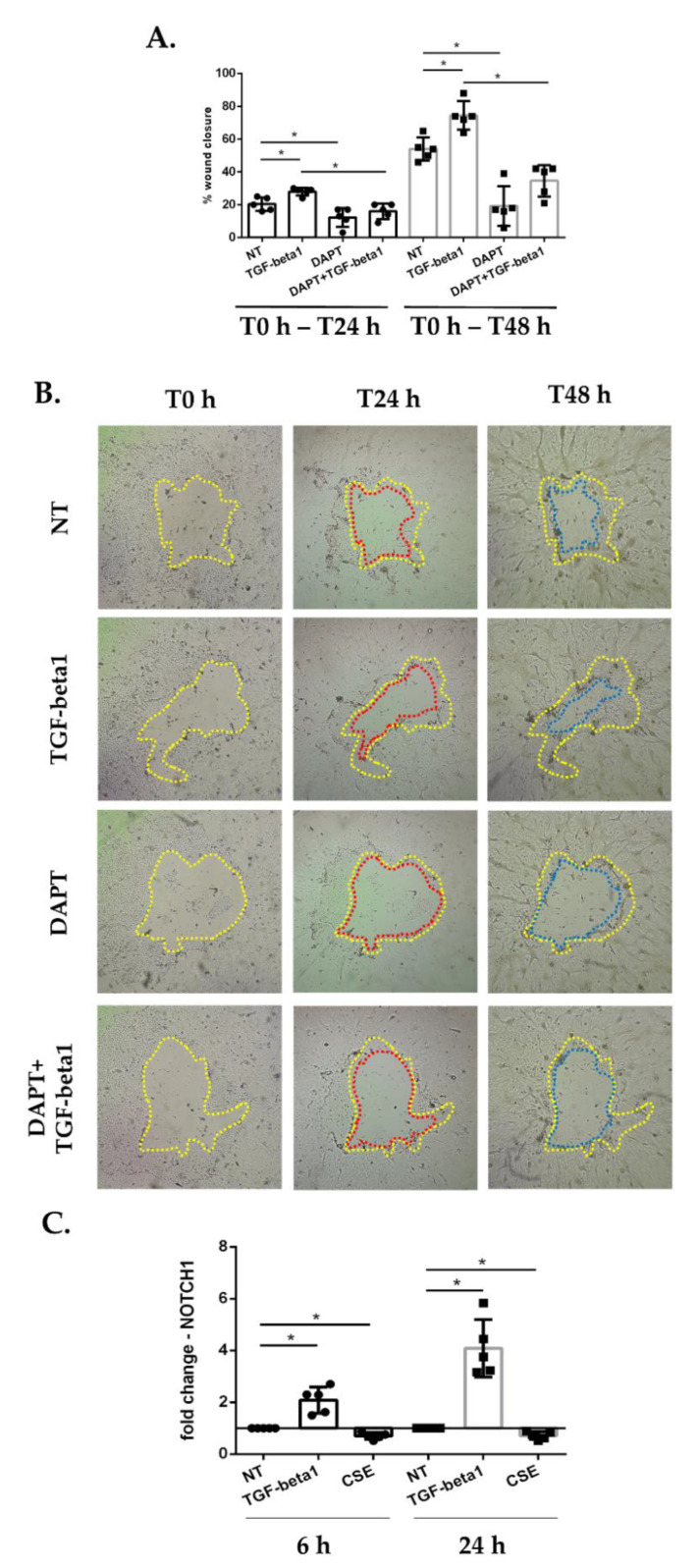
**Wound repair assay and NOTCH1 gene expression in submerged-PBECs.** (**A**,**B**) Sub-PBECs were seeded in a 6-well plate and were cultured to confluence. Three wounds were made in each well using a 200-µL pipette tip, then sub-PBECs were stimulated with TGF-beta1 and 10 μM DAPT. (**A**) Results are reported as percentage of area reduction at T24 h and 48 h compared to T0 h (*n* = 5). * *p* < 0.05 Mann–Whitney; (**B**) Representative images from one donor. The line colors indicate: yellow, the area of the wound at T0 h; red, the area at T24 h; blue, the area at T48 h; and (**C**) Sub-PBECs were stimulated with TGF-beta1 or CSE. After 6 h and 24 h, total RNA was extracted to evaluate NOTCH1 gene expression. Results are reported as mean fold change compared to not-treated (NT) ± SD (N = 5). * *p* < 0.05 Wilcoxon test.

In view of the role of the Notch pathway in wound repair [16], we evaluated the effect of Notch signaling inhibition on epithelial wound repair (Figure 3A,B) and the interference of this inhibition on activation of repair (in presence or absence of TGF-beta1). Submerged PBECs were treated with TGF-beta1 and/or 10 μM DAPT, a gamma-secretase inhibitor that blocks the Notch pathway, and wound repair was evaluated after 24 h and 48 h. The results showed that DAPT significantly delayed wound repair, even in presence of TGF-beta1 (Figure 3A,B).

As a follow-up to these findings, we evaluated whether exposure to cigarette smoke and TGF-beta1 had an effect on NOTCH1 gene expression. Our results showed that TGF-beta1 increased expression of NOTCH1, whereas CSE reduced its expression at 6 and 24 h following exposure (Figure 3C).

### 3.3. Cigarette Smoke Reduces E-Cadherin Gene and Protein Expression in ALI-PBECs

**Figure 4:**
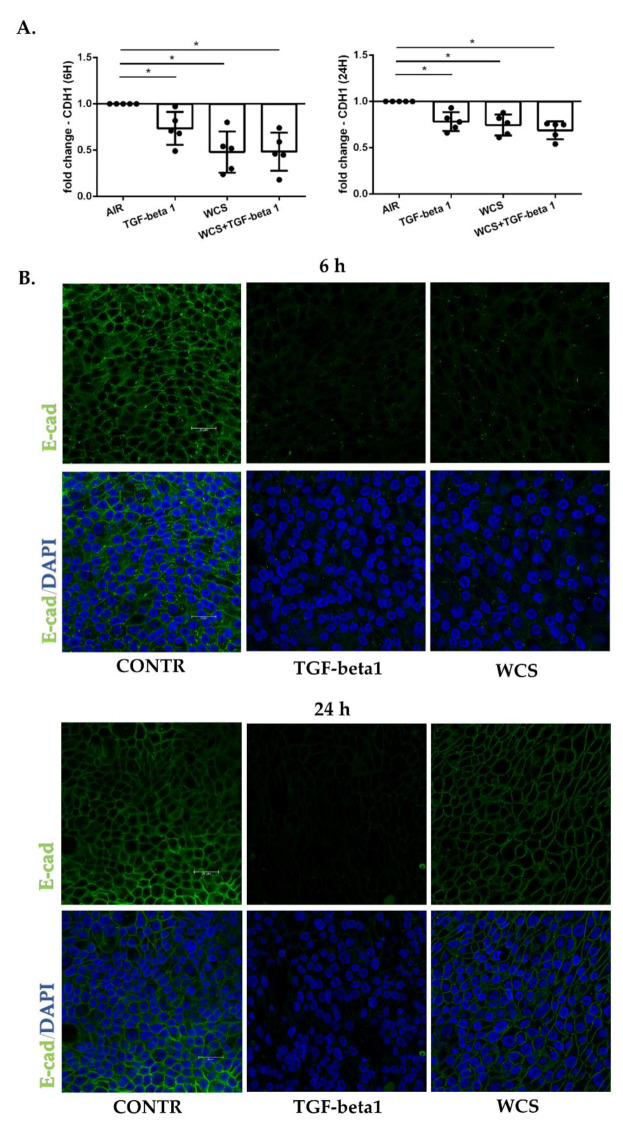
**E-cadherin gene and protein expression in differentiated ALI-PBECs**. PBECs were differentiated using ALI-culture and stimulated with TGF-beta1 and/or WCS. After 6 h and 24 h, total RNA was extracted and immunofluorescence staining was performed to evaluate E-cadherin gene (CDH1) and protein expression: (**A**) CDH1 gene expression. Results from 7 independent donors are reported as mean fold change compared to control (AIR) ± SD (*n* = 7). * *p* < 0.05 Wilcoxon test; and (**B**) Representative immunofluorescence staining for E-cadherin protein (green) in combination with 4′,6-diamidino-2-phenylindole (DAPI; blue) for nuclear staining. Scale bar, insert in control (CONTR) figure, is 25 µm for all images.

In order to validate our findings in a more complex experimental model that was closer to the cellular composition of the respiratory epithelium, PBECs were differentiated using air–liquid interface cultures (ALI-PBECs). ALI-PBECs were exposed to TGF-beta1 and/or whole cigarette smoke (WCS), and gene and protein expression of E-cadherin after 6 and 24 h were evaluated by RT-PCR and immunofluorescence, respectively. The results showed a decrease in gene expression of E-cadherin (Figure 4A) in all conditions. This reflected in the reduction of E-cadherin protein expression as reported in Figure 4B (Mean Gray Value 6 h: Control = 13734, TGF-beta1 = 4204, WCS = 4555; Mean Gray Value 24 h: Control = 11591, TGF-beta1 = 2731, WCS = 7404).

### 3.4. Cigarette Smoke Reduces Vimentin Gene and Protein Expression in ALI-PBECs

**Figure 5:**
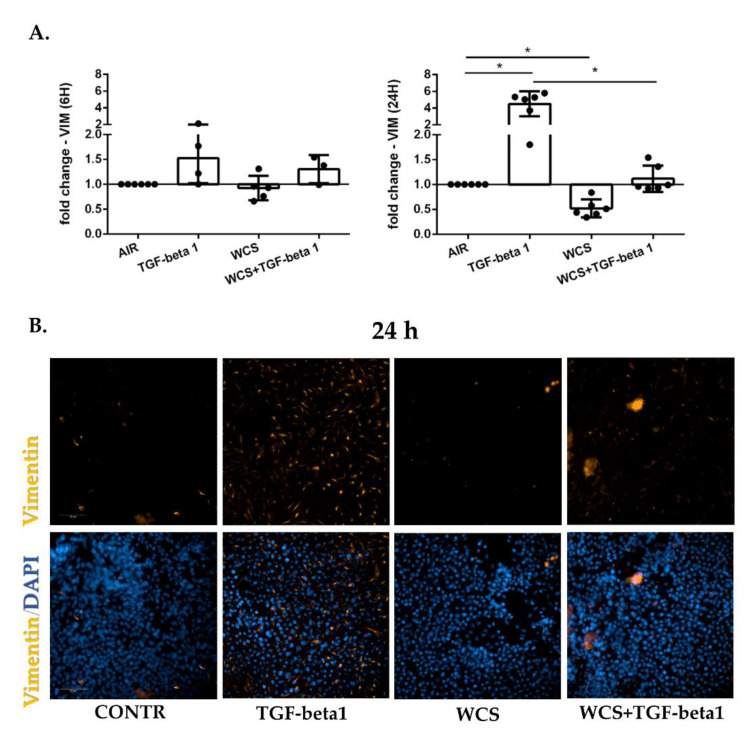
**Vimentin gene and protein expression in differentiated ALI-PBECs**. PBECs were differentiated using ALI-culture and stimulated with TGF-beta1 and/or WCS. After 6 h and 24 h, total RNA was extracted, and immunofluorescence staining was performed to evaluate Vimentin gene and protein expression: (**A**) VIMENTIN (VIM) gene expression. Results from 6 independent donors are reported as mean fold change compared to control (AIR) ± SD (*n* = 6). * *p* < 0.05 Wilcoxon test; and (**B**) Representative immunofluorescence staining for Vimentin protein (orange) in combination with 4′,6-diamidino-2-phenylindole (DAPI; blue) for nuclear staining. Scale bar, insert in CONTR figure, is 50 µm for all images.

ALI- PBECs were exposed to TGF-beta1 and/or WCS, and gene and protein expression of vimentin were evaluated after 6 and 24 h by RT-PCR and immunofluorescence, respectively. The results showed that after 6 h, TGF-beta1 caused a slight increase in vimentin gene expression, whereas WCS did not affect vimentin expression (Figure 5A). After 24 h, TGF-beta1 induced a stronger increase in vimentin gene expression, whereas WCS induced an opposite response leading to a decrease of vimentin expression (Figure 5A). The combination of TGF-beta1 and WCS did not alter VIMENTIN gene expression, and its expression was significantly different from that with TGF-beta1 alone after 24 h (Figure 5A). Figure 5B confirmed the increase of vimentin induced by TGF-beta1 and the decrease after exposure to WCS at protein level at 24 h. Since, in this case, the results on the expression of the vimentin gene showed that cigarette smoke interferes with the increase of vimentin due to TGF-beta1, we evaluated the effects of treatment with the WCS and TGF-beta1 combination also at the protein level. The results showed that exposure to TGF-beta1 in combination with WCS reduced the levels of vimentin protein compared to TGF-beta1 alone (Mean Gray Value 24 h: Control = 0.565, TGF-beta1 = 5573, WCS= 0.252, TGF-beta1 + WCS = 3429).

### 3.5. Cigarette Smoke Regulates the Gene Expression of EMT Transcription Factors in ALI-PBECs and in an ALI-PBECs/Fibroblasts Co-Culture Model

**Figure 6:**
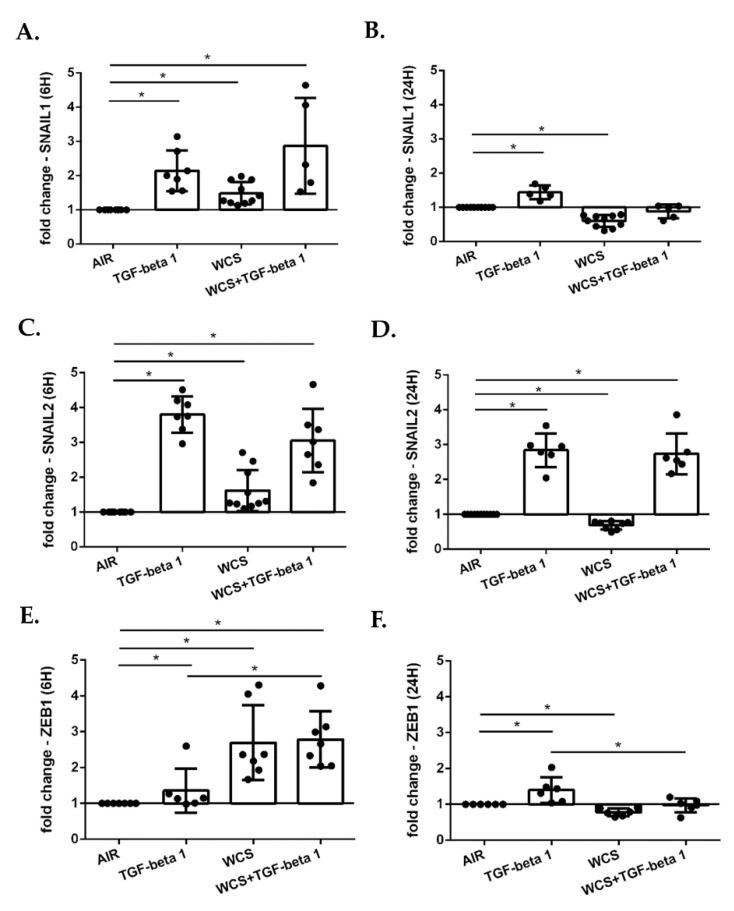
**SNAIL1, SNAIL2 and ZEB1 gene expression in differentiated ALI-PBECs**. PBECs were differentiated using ALI-culture and stimulated with TGF-beta1 and/or WCS. After 6 h and 24 h, total RNA was extracted to evaluate SNAIL1, SNAIL2 and ZEB1 gene expression. SNAIL1 gene expression after 6 h (**A**); and 24 h (**B**). SNAIL2 gene expression after 6 h (**C**); and 24 h (**D**). ZEB1 gene expression after 6 h (**E**); and 24 h (**F**). Results from 10 independent donors are reported as mean fold change compared to control (AIR) ± SD (*n* = 10). * *p* < 0.05 Wilcoxon test.

To further investigate the role of cigarette smoke on EMT in ALI-PBECs, we evaluated the gene expression of three transcription factors that regulates the EMT process: SNAIL1, SNAIL2 and ZEB1. The results showed that TGF-beta1, WCS and the combination of TGF-beta1 and WCS increased the gene expression of SNAIL1 (Figure 6A), SNAIL2 (Figure 6C) and ZEB1 (Figure 6E) after 6 h from the exposure. After 24 h, an increase in expression of these genes was still present in TGF-beta1-treated cells, whereas expression was lower in WCS-exposed cells (Figure 6B,D,F). Interestingly, upon exposure to the combination of TGF-beta1 and WCS, a decrease in SNAIL1 and ZEB1 but an increase in SNAIL2 expression was observed at 24 h (Figure 6B,D,F).

**Figure 7:**
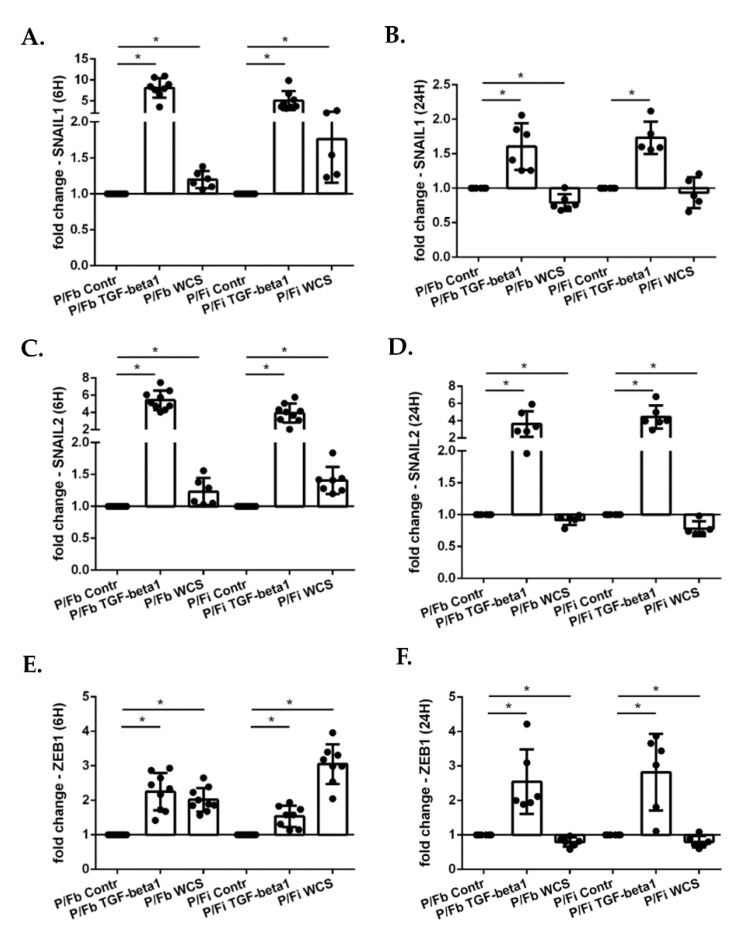
**SNAIL1, SNAIL2 and ZEB1 gene expression in co-culture model with ALI-PBECs and fibroblasts**. After two weeks of differentiation, ALI-PBECs were co-cultured with MRC5 fibroblasts. The fibroblasts were seeded on the bottom of the well (P/Fb) or on the inverted insert (P/Fi) where the differentiated ALI-PBECs were already present. After 24 h, the co-cultures were exposed to TGF-beta1 and/or WCS. After 6 h and 24 h of exposure, total RNA was extracted to evaluate SNAIL1, SNAIL2 and ZEB1 gene expression. SNAIL1 gene expression after 6 h (**A**); and 24 h (**B**). SNAIL2 gene expression after 6 h (**C**); and 24 h (**D**). ZEB1 gene expression after 6 h (**E**); and 24 h (**F**). Results from nine independent donors are reported as mean fold change compared to control (AIR) ± SD (*n* = 9). * *p* < 0.05 Wilcoxon test.

We next used a more complex experimental model in which ALI-PBECs are cultured together with MRC5 fibroblasts. We created two types of co-cultures: in the first we seeded the MRC5 fibroblasts on the bottom of the well of a trans-well with differentiated ALI-PBECs in the insert (P/F-b); in the second, the fibroblasts were seeded directly on the lower part of the insert membrane to assure that fibroblasts were in close proximity with the ALI-PBECs (P/F-i). Using these models, the effect of TGF-beta1 and WCS on gene expression of the transcription factors involved in EMT was evaluated. The results were similar to those obtained with the ALI-PBECs cultured alone (Figure 7): at 6 h, there was an increase of SNAIL1, SNAIL2, ZEB1 caused by TGF-beta1 and WCS (Figure 7A,C,E); at 24 h, TGF-beta1 induced an increase in expression of these gene, whereas WCS caused a decrease (Figure 7B,D,F).

### 3.6. Cigarette Smoke Decreases Gene Expression of MMP9 in ALI-PBECs and in the ALI-PBECs/Fibroblasts Co-Culture Model

**Figure 8:**
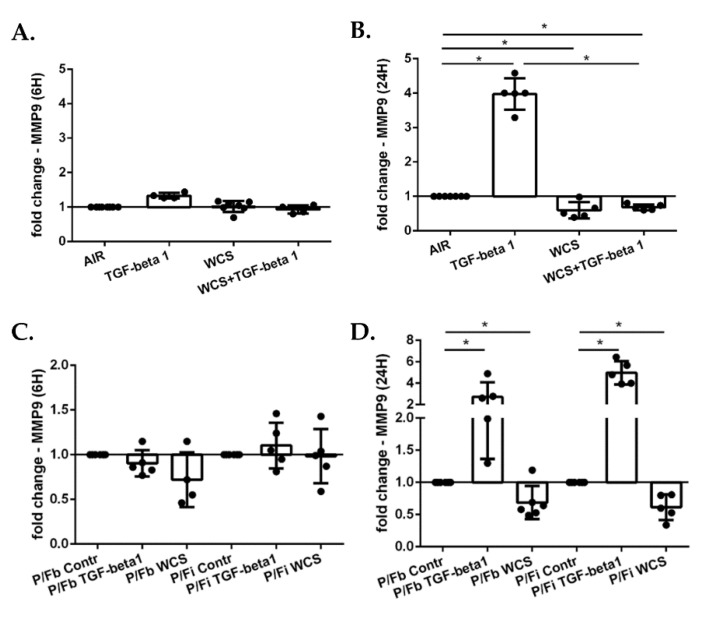
**MMP9 gene expression in differentiated ALI-PBECs and in co-culture model with ALI-PBECs and fibroblasts**. PBECs were differentiated using ALI-culture and stimulated with TGF-beta1 and/or WCS. After 6 h and 24 h of exposure, total RNA was extracted to evaluate MMP9 gene expression. MMP9 gene expression after 6 h (**A**); and 24 h (**B**). Results from 7 independent donors are reported as mean fold change compared to control (AIR) ± SD (*n* = 7). * *p* < 0.05 Wilcoxon test. After two weeks of differentiation, ALI-PBECs were co-cultured with MRC5 fibroblasts. The fibroblasts were seeded on the bottom of the well (P/Fb) or on the inverted insert (P/Fi) where the differentiated ALI-PBECs were already present. After 24 h, the co-cultures were exposed to TGF-beta1 and/or WCS. After 6 h and 24 h of exposure, total RNA was extracted to evaluate MMP9 gene expression. MMP9 gene expression after 6 h (**C**); and 24 h (**D**). Results from six independent donors are reported as mean fold change compared to control (AIR) ± SD (*n* = 6). * *p* < 0.05 Wilcoxon test.

The activation of EMT transcription factors is associated with the upregulation of matrix metallopeptidases (MMPs) such as MMP9, that is a critical protease facilitating cell migration during the EMT process [35]. Therefore, we evaluated the MMP9 gene expression after exposure to TGF-beta1 and WCS, alone and in combination. No changes were observed at 6 h (Figure 8A). In contrast, TGF-beta1 induced an increase in MMP9 gene expression at 24 h, whereas WCS induced a decrease (Figure 8B). When the ALI-PBECs were stimulated with both TGF-beta1 and WCS, the TGF-beta1-induced MMP9 expression was significantly blunted by WCS (Figure 8B). The same results were obtained in the co-cultures (Figure 8C,D).

### 3.7. Cigarette Smoke Decreases Gene Expression of TP63 and SCGB1A1 in ALI-PBECs and in the ALI-PBECs/Fibroblasts Co-Culture Model

**Figure 9:**
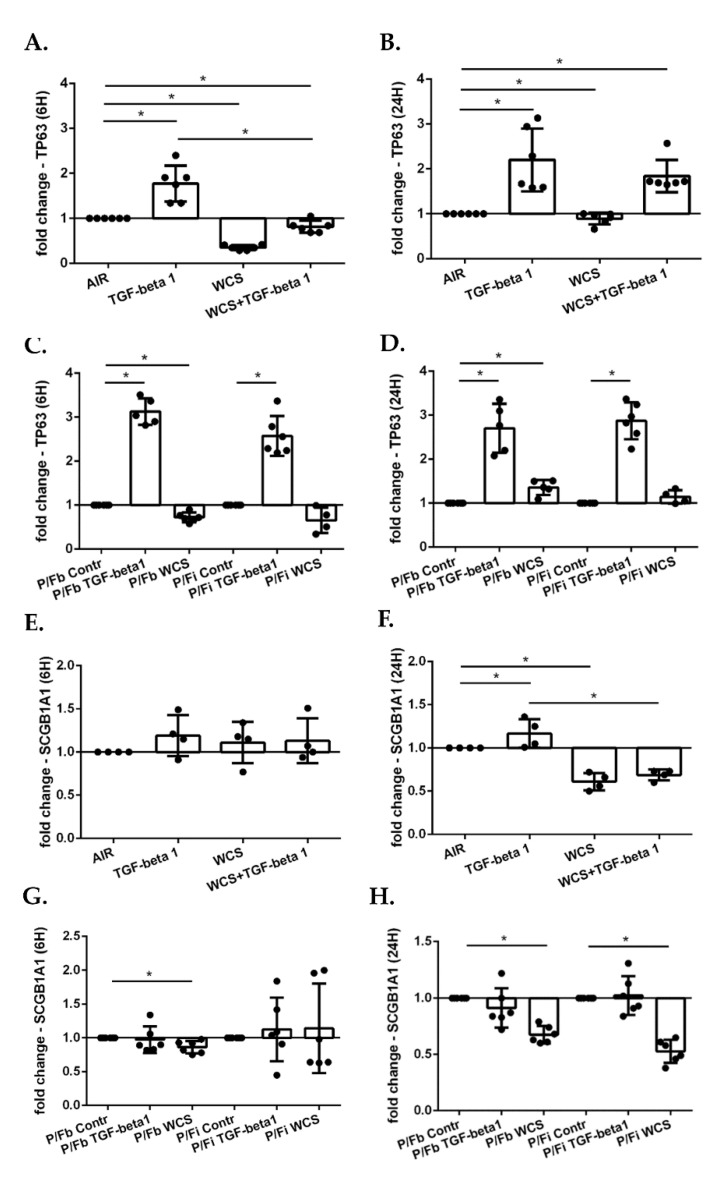
TP63 and SCGB1A1 gene expression in differentiated ALI-PBECs and in co-culture model with differentiated PBECs and fibroblasts. Panel (**A**,**B**,**E**,**F**). PBECs were differentiated using ALI-culture and stimulated with TGF-beta1 and/or WCS. After 6 h and 24 h of exposure, total RNA was extracted to evaluate TP63 and SCGB1A1 gene expression. TP63 gene expression after 6 h (**A**); and 24 h (**B**). Results from six independent donors are reported as mean fold change compared to control (AIR) ± SD (*n* = 6). SCGB1A1 gene expression after 6 h (**E**); and 24 h (**F**). Results from 4 independent donors are reported as mean fold change compared to control (AIR) ± SD (*n* = 4). * *p* < 0.05 Wilcoxon test. Panel (**C**,**D**,**G**,**H**). After two weeks of differentiation, ALI-PBECs were co-cultured with MRC5 fibroblasts. The fibroblasts were seeded on the bottom of the well (P/Fb) or on the inverted insert (P/Fi) where the differentiated ALI-PBECs were already present. After 24 h, the co-cultures were exposed to TGF-beta1 and/or WCS. After 6 h and 24 h of exposure, total RNA was extracted to evaluate TP63 and SCGB1A1 gene expression. TP63 gene expression after 6 h (**C**) and 24 h (**D**). Results from 6 independent donors are reported as mean fold change compared to control (AIR) ± SD (N = 6). SCGB1A1 gene expression after 6 h (**G**) and 24 h (**H**). Results from 4 independent donors are reported as mean fold change compared to control (AIR) ± SD (*n* = 4). * *p* < 0.05 Wilcoxon test.

TP63 is a marker of basal cells which serve as progenitor cells [36]. Our results showed that TGF-beta1 increased the relative expression of this gene at 6 h and 24 h (Figure 9A,B), whereas WCS caused a decrease. Upon combined exposure to TGF-beta1 and WCS, TP63 was lowered at 6 h (Figure 9A) and increased at 24 h (Figure 9B). Presence of fibroblasts in the two co-culture models did not markedly change the response of the ALI-PBECs at 6 h from exposure to TGF-beta1 and WCS (Figure 9C), whereas after 24 h TGF-beta1 increased TP63 gene expression, and WCS did not induce a decrease (Figure 9D).

We next also evaluated the expression of the club cell marker, SCGB1A1. The results showed that TGF-beta1 increased SCGB1A1 at 24 h, whereas WCS caused a decrease (Figure 9F). There was also a decrease of SCGB1A1 at 24 h after stimulation with the combination of TGF-beta1 and WCS (Figure 9F). We obtained similar results in the co-culture ALI-PBECs/Fibroblasts model (Figure 9G,H).

### 3.8. Cigarette Smoke Decreases the Gene Expression of NOTCH1, JAG1 and HES1 in ALI-PBECs 

**Figure 10:**
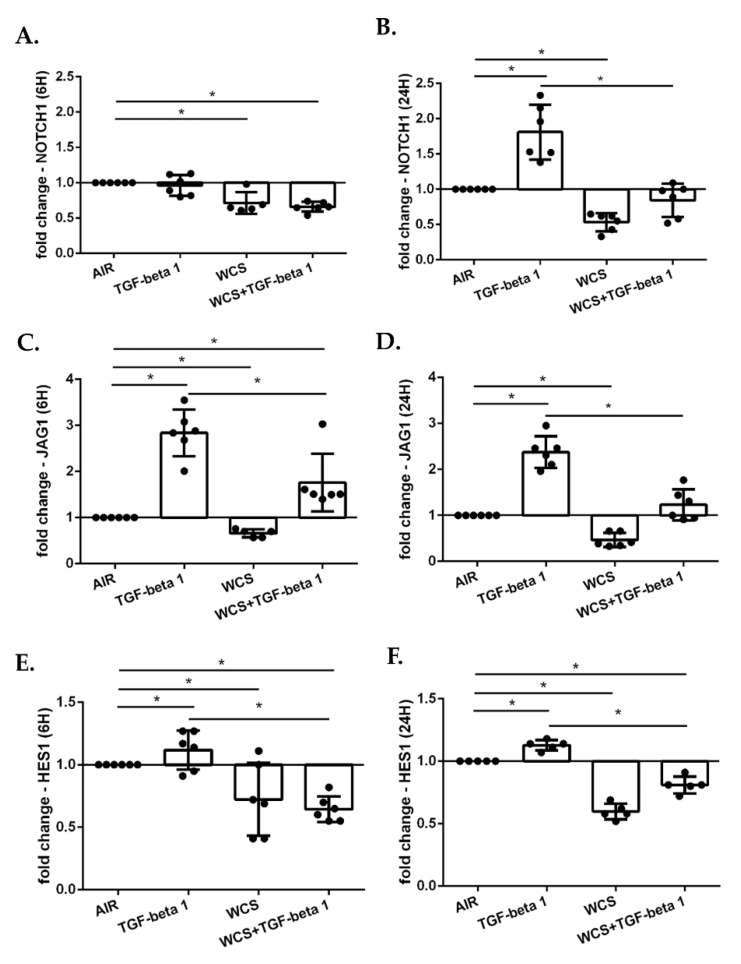
**NOTCH1, JAG1, HES-1 gene expression in differentiated ALI-PBECs**. PBECs were differentiated using ALI-culture and stimulated with TGF-beta1 and/or WCS. After 6 h and 24 h, total RNA was extracted to evaluate NOTCH1, JAG1, HES-1 gene expression. NOTCH1 gene expression after 6 h (**A**); and 24 h (**B**). JAG1 gene expression after 6 h (**C**); and 24 h (**D**). HES-1 gene expression after 6 h (**E**); and 24 h (**F**). Results from six independent donors are reported as mean fold change compared to control (AIR) ± SD (*n* = 6). * *p* < 0.05 Wilcoxon test.

We assessed whether cigarette smoke affected Notch-1 pathway also in ALI-PBECs. As reported in Figure 10, TGF-beta1 increased the gene expression of NOTCH1 (Figure 10A,B), JAG1 (Figure 10C,D) and HES1 (Figure 10E,F). On the contrary, WCS induced a decrease of the expression of these genes at 6 h and also at 24 h (Figure 10A–F). When cells were exposed to the combination of TGF-beta1 and WCS, the inhibitory effect of WCS dominated the stimulatory effects of TGF-beta1 (Figure 10A–F).

## 4. Discussion

Airway epithelial cells function as the first defense barrier against inhaled insults, such as pathogens and toxic substances, including cigarette smoke [37]. In case of breach of barrier integrity, a wound repair process is rapidly initiated. During this process, airway epithelial cells start EMT processes to repair the pseudostratified layer. Cigarette smoke *per se* or cigarette smoke-induced inflammation decreases wound repair [33]. Cigarette smoke is known to increase epithelial permeability, inflammation and tissue injury [33], through the induction of oxidative stress. However, it is not completely clear how cigarette smoke exposure modulates epithelial repair. Here, we provide evidence that cigarette smoke may interfere with the repair mechanisms of the bronchial epithelium by preventing the normal activation of TGF-beta1 induced-EMT processes and interfering with Notch-1 signaling (see Graphical abstract). 

Our study was conducted in three phases: in the first phase, we used a simple and convenient culture model in which undifferentiated primary bronchial epithelial cells, mainly consisting of basal cells, were grown in submerged culture. This model was used to study the effect of cigarette smoke on the repair of epithelial wounds created by scraping, and the role of the TGF-beta1 induced EMT and of the regulation of Notch-1 pathway. In the second phase, we used a more physiologically relevant model of mucociliary differentiated bronchial epithelial cells obtained by culturing the primary bronchial epithelial cells at the air–liquid interface. In this more complex in vitro model, the multiple epithelial cell types also observed in the airways are present, and therefore the model provides a better representation of airway tissue. The results obtained during this second phase, did not differ from those obtained from the submerged PBEC model, and provided us with more information on possible role of modulation of EMT and Notch signaling by TGF-beta1 and cigarette smoke in wound repair. In the third phase of the study, we added further complexity to the model by including fibroblasts, allowing us to study the involvement of epithelium-fibroblast crosstalk on epithelial repair and EMT. In view of the increased insight into the substantial heterogeneity of fibroblast populations in different parts of the lung, we have chosen to use a cell line of fibroblasts in order not to increase the experimental variability. The results showed that addition to the fibroblasts did not modify the observed effects of TGF-beta and cigarette smoke on the components of these pathways. It needs to be noted, however, that also the fibroblast-ALI-PBEC co-culture has important limitations, also because in lung tissue various other cell types and mediators are present. 

Our results show that cigarette smoke does induce the initial steps of EMT, but blocks later EMT events (including acquisition of mesenchymal markers). It is tempting to speculate that this modulation of EMT contributes to smoke-induced impairment of wound repair, but whether and how this occurs requires further detailed investigation. The first step in the initiation of EMT is the destabilization of adherens junctions by downregulation of E-cadherin, accompanied by the increase of N-cadherin [10,38]. Our results showed that in primary bronchial epithelial cells, both when cultured submerged and when differentiated using ALI culture, cigarette smoke caused a decrease in E-cadherin without a concomitant increase in N-cadherin (data not shown) at 6 h after the exposure. We found a more limited decrease of E-cadherin at later time points (24 h) after exposure to cigarette smoke, and a decrease of N-cadherin (data not shown). The subsequent phase of the EMT process is characterized by an increase in expression of genes encoding cytoskeletal and polarity complex proteins, which are characteristic of a mesenchymal cell phenotype [10,39,40]. Whereas in our in vitro model, TGF-beta1 increased expression of the mesenchymal marker vimentin, cigarette smoke exposure reduced TGF-beta1-induced vimentin expression. These results demonstrate that exposure to cigarette smoke interferes with the acquisition of mesenchymal cell phenotype, one of the steps in wound repair.

To better explain these differences, we assessed simultaneously the impact of TGF-beta1, cigarette smoke or their combination, on expression of three transcription factors involved in EMT regulation, SNAIL1, SNAIL2 and ZEB1. SNAIL1 and SNAIL2 repress epithelial genes, including E-cadherin, by binding to E-box DNA sequences through their carboxy terminal zinc finger domains [41,42]. When SNAIL1 binds to the promoter of the E-cadherin gene, it recruits a protein complex that coordinate histone modifications, specifically methylation and acetylation, resulting in transient repression of E-cadherin gene expression. The nuclear localization and degradation of Snail1 and Slug (the products of the genes SNAIL1 and SNAIL2) is controlled by several mechanisms, including post-translational modifications. Phosphorylation of specific amino acids in the Snail proteins by p21-activated kinase (PAK1), casein kinase 1 (CK1) and glycogen synthase kinase 3β (GSK-3β) promotes their proteasomal degradation [10,43,44]. Furthermore, p53 induces the expression of miR-34 that suppresses SNAIL1 expression, and promotes the degradation of Slug through the ubiquitin ligase murine double minute 2 (MDM2) in a ternary complex of p53, Slug and MDM2 [44,45,46]. ZEB1 also mediates a transcriptional repression of the E-cadherin gene by binding the E-box of the promoter and recruiting a co-repressor complex [47]. The ZEB1 gene is a target of SNAIL1, and therefore an increase in ZEB1 expression follows SNAIL activation [48]. Expression of ZEB1 is also controlled by other mechanisms, including post-transcriptional modifications as well as the action of some microRNAs such as miR-200 [49,50]. In the present study we evaluated the gene expression of these transcription factors and the results obtained confirm the interference of cigarette smoke in the complete activation of the EMT process. In fact, within a few hours after cigarette smoke exposure there was an increase in the expression of SNAIL1, SNAIL2 and ZEB1, but the results showed a decrease in these transcription factors at 24 h. On the other hand, TGF-beta1 induced an early increase in SNAIL1, SNAIL2 and ZEB1 that persisted at 24 h. Interestingly, stimulation with TGF-beta1 following cigarette smoke exposure for 24 h, was found to prevent the complete activation of this mechanism.

Several studies demonstrated that cigarette smoke decreases E-cadherin in mice [51,52], human bronchial epithelial cell lines (16HBE, BEAS-2B) [53,54] and also in primary bronchial epithelial cells isolated from proximal [55] and small bronchi [56,57]. Other studies showed deregulation of EMT biomarkers in tissue sections from smokers, smokers with COPD and ex-smokers with COPD [58,59]. Several authors have shown that the EMT process is different in large and small airways. Mahmood et al. [58] found that in the small airways there is predominantly a type 2 EMT with the development of fibrotic tissue, while in the large airways there is a type 3 EMT which may help to explain the high frequency of tumors related to COPD. However, in all these studies, patients had a history of decades of cigarette smoke exposure. In contrast, in our study we evaluated, for the first time, the effects of acute cigarette smoke exposure showing a dual response of the injured epithelium: immediately after the cigarette smoke exposure, some markers of EMT are initially induced but in an incomplete manner (i.e., without inducing vimentin expression) at 24 h. This aberrant activation of EMT could be linked to a decreased repair response. It is possible to argue that cigarettes smoke also activates mechanisms that limit the expression of transcription factors related to EMT. For this reason, we evaluated miR-200c expression which represses ZEB1 expression [9,49] in ALI-PBECs, but our results showed that WCS has no effect on this factor (data not shown). Cigarette smoke could interfere with other post-translational modification mechanisms and this could be evaluated in future studies.

The relevance of our findings is underscored by the fact that they were obtained using primary bronchial epithelial cells from 11 different donors, and that these were differentiated to form a pseudostratified epithelium. Furthermore, the exposure to WCS creates in vitro a condition that is reminiscent to the in vivo situation in the lung of smokers, resulting in exposure to both the particulate and the gaseous phase of cigarette smoke. Similar findings were obtained when fibroblasts were included in the experimental model using co-culture with ALI-PBECs in an attempt to recreate the cellular environment of the epithelial cells in airways. Several studies show that chronic inflammation and remodeling of the airways and parenchyma, events that are a characteristic of a variety of respiratory diseases, occur due to aberrant activation of the “epithelial-mesenchymal trophic unit” (EMTU), a structural and functional element in the lung [60] in which there is a central role for crosstalk between the pulmonary epithelium and the fibroblasts. The epithelium responds locally to the insult through the release of various epithelial inflammatory mediators and growth factors, and this modifies the extracellular matrix and influences the cellular phenotype of the fibroblast sheath. In turn, fibroblasts influence the phenotype of the airway epithelium [60,61].

Furthermore, we also explored other mechanisms involved in epithelial repair. MMPs are important in the repair of epithelial wounds as they degrade the components of the extracellular matrix by provisional remodeling of the site where cells move [62]. MMP9 plays a key role in the migration of bronchial epithelial cells involved in wound repair and it is overexpressed in these cells during the re-epithelialization process [37]. Our data showed that cigarette smoke reduces the expression of MMP9 and also in this case it counteracts the increase due to TGF-beta1. Whether this may lead to inhibition of epithelial migration into the wound area in the early phase of repair following smoke exposure [33] will require further investigation.

Our findings support the notion that cigarette smoke reduces the reparative potential of the airway epithelium and may alter its cellular composition. In this regard, we found a decreased expression of TP63 suggesting a reduction of basal progenitor cells, that have a relevant role in the repair of the damage and in restoring epithelial integrity and tissue homeostasis. In addition, cigarette smoke alters tissue homeostasis negatively by interfering with differentiative processes as demonstrated by the reduced numbers of club cells suggested by decreased SCGB1A1 expression.

Another important actor in the physiologic repair process is the Notch pathway. For this reason, we tested the effect of blocking the Notch pathway, using the inhibitor DAPT, on epithelial wound repair. Results herein reported showed that DAPT decreases repair. This finding led us to hypothesize that the impact of cigarette smoke on wound repair could be in part mediated by inhibition of Notch signaling by cigarette smoke. Consistent with this hypothesis, cigarette smoke exposure reduced Notch-1 expression in all experimental models used in the present study. Hence, our findings suggest that cigarette smoke impairs epithelial wound repair by interfering with EMT processes and reducing the expression of components of the Notch signaling pathway. Furthermore, Notch signaling also participates in developmental EMT and contributes to the generation of cancer stem cells [63]. In the respiratory epithelium, basal cells (TP63+) are the stem cells of adult tissue that self-renew and through differentiation mechanisms generate specialized luminal cells [36,64,65]. The activation of the Notch pathway is required for the differentiation of the basal cells and promotes the secretory lineage with the increase of club cells (SCGB1A+) and goblet cells [64]. Our data showing a reduction of SCGB1A1 expression may be associated with the reduced functionality of the Notch pathway. A previous study by our group has demonstrated a defective nuclear expression of Notch-1 protein in bronchial epithelium of smokers and has provided data showing that, in the bronchial epithelial cell line 16HBE, cigarette smoke extract reduces nuclear translocation of Notch-1, a phenomenon with a relevant role in modulating Notch-1 target gene expression [27]. In this regard, it has been demonstrated that during lung embryogenesis a defective Notch signaling activation in proximal airways leads to a preferential commitment of airway proximal progenitors cells toward ciliated cells rather than toward secretory cells [66], a findings that has been widely recapitulated in cell culture models including our own [30]. Furthermore, since the Notch pathway is implicated in EMT by regulating SNAIL1 and SNAIL2 gene expression [23,24], it would be interesting to explore the link between Notch, EMT and repair mechanisms in relation to cigarette smoke in subsequent studies.

## 5. Conclusions

This study provides compelling evidence that the acute insult exerted by cigarette smoke leads to activation of an abortive repair program in proximal airway epithelial cells. In line with this we observed that cigarette smoke counteracts the reparative effects exerted by TGF-beta1, negatively affecting the restoration of tissue homeostasis in the event of damage to the airways. 

Further studies will be required to unravel the role of the Notch-1 pathway, to understand at what level and how cigarette smoke disrupts EMT by, e.g., studying the nuclear protein expression of EMT transcription factors (Snail1, Slug and ZEB1) at different time points, and by delineating the mechanisms that regulate their nuclear translocation or degradation. It will next be important to investigate the functional consequences of this interference in the repair and the role of oxidative stress. A better understanding of these processes is fundamental to understand the development and progression of smoking-related lung diseases, and will also have possible important implications for understanding the impact of air pollution. 

## Figures and Tables

**Table 1:** Primers sequences.

Gene Name	Protein Name	Forward Sequence (5’-->3’)	Reverse Sequence (5’-->3’)	Accession Number (GenBank)
CDH1	E-cadherin	TTCCCAACTCCTCTCCTG	AAACCTTGCCTTCTTTGTC	NM_001317185.2; NM_001317186.2; NM_001317184.2; NM_004360.5
VIMENTIN	Vimentin	TTGAACGCAAAGTGGAATC	AGGTCAGGCTTGGAAACA	NM_003380.5
ZEB-1	ZEB-1	TTACACCTTTGCATACAGAACCC	GATGATGAATGCGAGTCAGATGC	NM_001128128.3
SNAIL1	Snail1	TCGGAAGCCTAACTACAGCGA	AGATGAGCATTGGCAGCGAG	NM_005985.4
SNAIL2	Slug	TGTGACAAGGAATATGTGAGCC	TGAGCCCTCAGATTTGACCTG	NM_003068.5
TP63	TP63	CCACCTGGACGTATTCCACTG	TCGAATCAAATGACTAGGAGGGG	NM_001329964.2
SCGB1A1	SCGB1A1	ACATGAGGGAGGCAGGGGCTC	ACTCAAAGCATGGCAGCGGCA	NM_003357.5
RPL13A	RPL13A	AAGGTGGTGGTCGTACGCTGTG	CGGGAAGGGTTGGTGTTCATCC	NM_012423.4
ATP5B	ATP5B	TCACCCAGGCTGGTTCAGA	AGTGGCCAGGGTAGGCTGAT	NM_001686.4

## Data Availability

The data presented in this study are available in the article.
